# Stability of a Cosmetic Multiple Emulsion Loaded with Green Tea Extract

**DOI:** 10.1155/2013/153695

**Published:** 2013-08-24

**Authors:** Tariq Mahmood, Naveed Akhtar

**Affiliations:** Department of Pharmacy, Faculty of Pharmacy and Alternative Medicine, The Islamia University of Bahawalpur, Bahawalpur 63100, Pakistan

## Abstract

Multiple emulsions are excellent and exciting potential systems for the delivery of useful cosmetic agents. The work describes stability of a multiple emulsion for cosmetic purpose, loaded with extract of *Camellia sinensis* L. (Theaceae) in concentration of 5%. The formulation constitutes of cetyl dimethicone copolyol and polyoxyethylene (20) cetyl ether as emulsifiers and was characterised and monitored for various physicochemical aspects. Centrifugation has no devastating effect on physical destabilization/phase separation observed for 30 days. Mean globule sizes of multiple droplets were found in the range of 10.29 ± 4.4 **μ**m to 12.77 ± 5.1 **μ**m and of inner droplets were in the range of 0.8 ± 0.4 **μ**m to 1.6 ± 0.8 **μ**m. All samples exhibited shear thinning behavior with increase in shear stress. The results of the present study indicate that multiple emulsions can be used as carrier of 5% *Camellia sinensis* L. extract to enhance desired effects. The developed physically and chemically stable system is an effective system for targeting skin layers; however, long-term stability at elevated temperatures may be needed with suitable modifications, if required.

## 1. Introduction

The emulsion was discovered as long ago as 1925 [1]. Emulsions are dispersed multiple phase systems. They are made out of at least two nearly immiscible fluids, one being dispersed in the other. The dispersed phase forms droplets, which are surrounded by the continuous phase [2]. Multiple emulsions are very complex systems [3]. These are characterized by the vesicular structures, where internal and external aqueous phases are separated with oil membranes. Owing to the liquid membrane structures of oil phases, W/O/W emulsions have many potential applications in such diverse fields as pharmaceutics, cosmetics, food, and separation technologies [1].

Due to their unique properties and structures, multiple emulsions are interesting carrier systems for various drug delivery approaches [4]. However, compared with simple emulsions consisting of only two phases, much more destabilization processes need to be taken into consideration for multiple emulsions [5]. In cosmetics, multiple emulsions are useful when one wishes to prepare sustained release aerosol fragrances, prolonged skin moisturizers and protection of sensitive biologicals, personal care formulations for perfumes, skin lipids, vitamins, and free radical scavengers [6].

Cosmeceuticals are discussed internationally as they contain “bioactives” which, though not medicinal, are gifted with useful and quantifiable attributes. Alternative terms for cosmeceuticals have sprung up (performance cosmetics, functional cosmetics, dermaceuticals, and active cosmetics) [7].

The current study is projected to develop and characterize a stable cosmetic multiple emulsions loaded with 5% *Camellia sinensis* L. extract; thus, it could be explored for its cosmetic effects in novel carrier systems.

## 2. Materials and Methods

### 2.1. Materials

Multiple emulsions were developed using the following chemicals: liquid paraffin (*ή*: 110–230 mPa·s, Merck, Germany). The lipophilic emulsifier cetyl dimethicone copolyol (Abil EM 90) was kindly provided by Franken (Franken, Germany). Polyoxyethylene (20) cetyl ether (Brij 58) used as hydrophilic emulsifiers and supplied by (Merck, Germany). MgSO_4_·7H_2_O was used as conductometric tracer in the inner aqueous phase of the primary emulsion. Standardized green tea extract (5%) was encapsulated as “green cosmetic agent” in the inner phase of the primary emulsion and was extracted in our lab. All materials were of analytical grade and used without further purification.

### 2.2. Preparation of W/O/W Type Multiple Emulsions

Several preformulation studies [8] helped us in the selection of the most appropriate primary emulsion for the further development of W/O/W multiple emulsion system. Composition of the selected emulsion is shown in Table 1. All multiple emulsions were produced by two-step emulsification procedure [3]. Briefly, primary emulsion was prepared by emulsifying the oil phase with the aqueous phase in the presence of lipophilic surfactant while heating both phases at 75°C in a digital water bath (Heidolph, Germany). Green tea extract and the conductimetric tracer were incorporated into the aqueous internal phase of the primary emulsion. Homogenization of the aqueous phase with the oil phase was achieved with an IKA Mixing Overhead Stirrer, Eurostar (IKA, Werke, Germany) at 2000 rpm for 10 minutes and then reduced to 1000 rpm for 10 minutes, and finally the emulsion was cooled to room temperature while maintaining a stirring speed of 500 rpm for further 10 minutes. The primary emulsion obtained was subjected to second stage emulsification in which the primary emulsion was added slowly to the aqueous phase containing hydrophilic emulsifier at stirring speed of 700 rpm for 60 minutes; at this time multiple emulsions were confirmed by microscopy. 

### 2.3. Stability Studies

Accelerated stability studies in the cosmetic area have been performed in periods of 30 to 90 days [9]. For stability studies, samples were weighed (50 g) and packed in glass containers with 100 g of content capacity. Samples were kept in incubation chambers, each preset at different storage temperature for a period of 30 days and this testing was called “the accelerated storage period” which is the time between the preparation of multiple emulsion and its physicochemical investigation. Glass containers were chosen to accelerate the conditions and hence to accelerate the destabilization of multiple emulsions. Different storage conditions were room temperature (25 ± 1°C), low temperature (8 ± 1°C), high temperature (40 ± 1°C), and high temperature with humidity (40 ± 1°C with 75% relative humidity). At the predetermined intervals (24 h, 48 h, 7 d, 15 d, and 30 d), samples were removed from the storage conditions and allowed to achieve room temperature (25°C) prior to the evaluation of the physicochemical characteristics.

### 2.4. Characterization of Green Tea Multiple Emulsion

After preparation, the multiple green tea multiple emulsion was characterized regarding its microscopic aspects, conductivity, pH, phase separation, and rheological behavior, and samples kept in different storage conditions (8°C, 25°C, 40°C, and at 40°C with 75% relative humidity) were followed for a period of 30 days. Before any observation for different storage conditions, samples were allowed to return to room temperature. 

 The specific data were evaluated using the statistical tool SPSS version 12.0 according to one-way ANOVA test defining a 5% level of significance. Standard deviation was calculated for every mean value. A statistical Grubb's test for detecting outliers was also performed on data by using following equation:

(1) $$\begin{array}{c} Z=\frac{\left|\text{mean-value}\right|}{\text{SD}}. \end{array}$$

### 2.5. Microscopic Analysis

Multiple characteristics of the prepared emulsions were determined via microscopic analysis, through an optical microscope (Nikon E200, Nikon, Japan) with a camera (DCM-35 USB 2.0 and Minisee Image software). Samples were observed at 100x magnification after diluting the samples with the external phase of the emulsion. Droplet diameter assessment of 30 droplets per sample per storage condition was performed with Digimizer, Version 4.1.1.0 (MedCalc Software, Mariakerke, Belgium). Mean droplet diameter was calculated for the droplets of samples kept in different storage conditions.

### 2.6. Conductimetric Analysis

Conductimetric analysis of undiluted samples was performed to examine the release of the electrolyte initially entrapped in the internal water phase. The specific conductivity of the emulsions was measured directly using a Digital Microprocessor Conductivity Meter (WTW-Tetracon, Germany) at 25 ± 2°C. Conductivity tests were performed for multiple emulsion formulation immediately after preparation and at 24 h on days 07, 15, and 30 for samples kept in different storage conditions. 

### 2.7. pH Determination

The pH of fresh sample and samples kept in different storage conditions was determined by a digital pH meter ProfiLine pH 197 (WTW, Germany). The pH measurements were also taken for the samples at 24 h on days 7, 15, and 30. 

### 2.8. Centrifugation

The emulsions were centrifuged at 25°C (3) with a centrifuge machine (Hettich EBA 20, Germany) at 5000 rpm for 20 minutes. Exaggerated centrifugation tests (ECTs) were performed for each sample at different time intervals (24 h on dayd 7, 15 and 30) kept in different storage conditions (08 ± 1°C, 25 ± 1°C, 40 ± 1°C, 40 ± 1°C + 75% RH). 

### 2.9. Rheological Examination

The viscosities of the samples were determined at 25°C spindle speeds ranging from 100 to 200 rpm while using a spindle CP 41 in a Brookfield programmable rheometer (Model DV.III; Brookfield engineering laboratories Inc., USA). Rheocalc V 2.6 (Microsoft Corporation) software was used as a support program to produce the results of rheological behavior.

## 3. Results and Discussion

In the development of multiple emulsions, the following variables are important and should be considered: (i) primary W/O emulsifier, usually a low HLB number surfactant, (ii) nature of the oil phase—various paraffinic oils are preferred, (iii) secondary emulsifier, that is, high HLB number polymer or surfactant, (iv) secondary volume fraction, that is, between 0.4 and 0.8 depending on required viscosity, (v) nature of electrolyte, (vi) thickeners or additives, and (vii) processing of primary emulsion at high shear mixing while of secondary emulsion at low shear mixing [5].

Multiple emulsion was developed using Abil EM90 as lipophilic emulsifier and liquid paraffin as oil phase. The electrolyte used was hydrated MgSO_4_ while Brij 58 was used as hydrophilic emulsifier in second stage emulsification. It was aimed to develop multiple emulsions without any additive or thickener. For this purpose, several pre-formulation studies were performed to obtain a final formulation with appropriate cream consistency just like thick creams of W/O type or O/W type. It was found that low-viscous primary emulsions with 2.5% Abil EM90 without any thickener in internal aqueous phase of the primary emulsion lead to the formation of more viscous multiple emulsions on secondary emulsification with a hydrophilic emulsifier Brij 58. It was also concluded that Brij 58 at high concentrations up to 5% has no destabilizing effects; rather, this concentration produced more consistent viscous multiple emulsions. Results, however, are different from previous studies which demonstrate that, irrespective of the type of surfactant, emulsion viscosity decreases as the concentration of hydrophilic emulsifier is increased. Not only the type of surfactant but the concentration thereof is therefore of importance when it comes to preparing multiple emulsions. It should be high enough to stabilize the O/W interface. The destabilizing effect must be taken into consideration, however, at higher concentrations [5].

MgSO_4_ (hydrated) was selected and incorporated as an electrolyte in the inner aqueous phase of the primary emulsion because NaCl can contribute to the viscosity of the system as well as it may lead to destabilization of systems during storage as reported previously [1].

Another important factor is mixing speed of both primary emulsification as well as secondary emulsification. As mentioned above, processing of primary emulsion should be performed at high shear mixing while of secondary emulsion at lower shear mixing as high shear mixing may affect globule size. Primary emulsion processing was performed at different mixing speeds. Firstly, a high shear mixing was provided to the primary emulsion at 2000 rpm for 10 minutes, then an intermediate speed of 1000 rpm for further 10 minutes and at the end lowering the speed to 500 rpm so that the emulsion could be cooled to room temperature. Secondary stage mixing speed was kept at 700 rpm until multiple emulsions were formed and were confirmed with optical microscopy. This experimentation led to conclude that process parameters in multiple emulsions are highly sensitive and should be strictly monitored in order to produce consistent and reproducible multiple emulsions. Even small variations could lead to formulations with different consistencies and ultimately with different characteristics of stability.

### 3.1. Microscopic Analysis and Centrifugation

Microscopic and globule size analysis is a quite useful direct method to assure formation of multiple emulsions as well as to predict stability of multiple globules over time. Droplet size measurements are a good indicator of the formulation stability.

Microscopic analysis was carried out during the development stage of multiple emulsions under the microscope to confirm multiple characteristics. Globule size measurements were followed for freshly prepared samples as well as for samples stored in different conditions of storage (8°C, 25°C, 40°C and at 40°C with 75% relative humidity) to observe any change in globule size over time. The size of droplets is an important parameter in the physical stability of an emulsion. Microscopic analysis was performed after every centrifugation cycle to determine multiple characteristics after exaggerated centrifugation test to confirm whether multiple globules are resistant to centrifugation or not. Various samples kept at different storage conditions were centrifuged at 5000 rpm for 20 minutes every time at 24 h, 48 h on days 7, 15, and 30, in order to accelerate the phase separation as well as to obtain droplet rich upper layer in case phase separation occurs.

**Figure 1:**
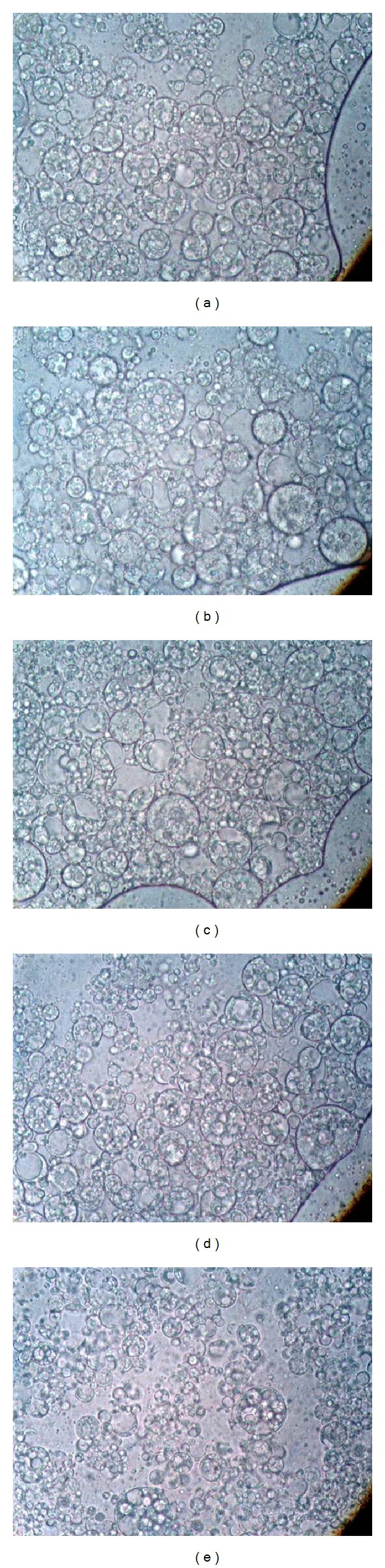
Photomicrographs of multiple emulsions kept at different storage conditions. (a) Fresh sample, (b) 8°C, (c) 25°C, (d) 40°C, (e) 40°C + 75% relative humidity.

Results of the investigation reveal that droplet size of multiple globules did not change significantly at any condition of storage, and also no physical destabilization/phase separation was observed after exaggerated centrifugation test (ECT), even throughout the followed period of 30 days. Photomicrographs of multiple emulsion samples kept in different conditions of storage are shown in Figure 1.

In this study, mean globule sizes of multiple droplets and inner droplets in multiple emulsions were recorded separately in order to correlate inner droplets size influence on the size of multiple emulsion droplets. The mean globule size of multiple droplets was recorded in the range of 10.29 ± 4.4 *μ*m to 12.77 ± 5.1 *μ*m (mean ± standard deviation), while globule size of inner droplets of multiple emulsions was recorded in the range of 0.8 ± 0.4 *μ*m to 1.6 ± 0.8 *μ*m (mean ± standard deviation). One-way ANOVA statistical test was applied to check any deviation in globule size in different conditions of storage. Statistically nonsignificant differences were observed (*P* > 0.05) regarding differences in globule sizes (multiple droplets and inner droplets) of samples kept in different conditions of storage. Grubbs' test was also applied, also called the ESD method (extreme studentized deviate), to determine whether one of the values in the list is a significant outlier from the rest. It was found only one value in each list which was far away from the rest but statistically nonsignificant outliers were recorded for each list (*Z* > 0.05). This test in terms validated the results of software package used to record globule sizes. Moreover, the incorporation of green tea extract may have an effect on emulsion droplet size due to coalescence of globules and decreased surface area. 

As none of the phenomena affected the globule sizes, no significant decrease in viscosity was observed as well during varying storage time and conditions. A correlation between globule size of inner droplets and multiple droplets was also found. It was observed that as the size of inner droplets increases, the size of multiple droplets also increases in parallel fashion. This phenomenon is also described previously, as it has been stated that one parameter to influence stability is droplet size, and smaller droplet size is always advantageous because reducing the droplet size leads to better emulsion stability against gravitational separation as Stoke's law describes. According to this information the ideal droplet size for inner droplets in W/O/W emulsion should be less than 1 *μ*m; however, oil droplets must be considerably greater in size, up to several *μ*m [10]. Records of microscopic analysis are shown in Table 2.

### 3.2. Conductimetric and pH Analysis

Conductimetric analysis was carried out to directly measure the entrapped conductimetric tracer in the inner aqueous phase of the primary emulsion. It was also an indicator of the active substance entrapped in the inner phase of the primary emulsion. The more a conductimetric tracer is released, the more the active substance is free to move in external aqueous phase, and thus the less sustained the effects will be. It is believed that during storage time conductivity values increase, and this phenomena is ascribed due to (i) diffusion of an electrolyte, (ii) the coalescence of internal and aqueous phases, and (iii) destruction of oil film because of osmotic pressure and the leakage of internal aqueous phase [11, 12]. When mean conductivity values were calculated, it was obvious that only at cold temperature the conductivity values were higher than the value of fresh sample. This may be due to leakage of internal aqueous phase to the external aqueous phase by any of the above explained mechanisms, but we did not observe any correlation of this increase on multiple droplet size or inner droplet size as well as on rheological behavior of the multiple emulsion samples. The increased conductivity values of formulation samples kept at different conditions may be attributed to entrapping a 5% green tea extract. It has been reported previously that conductivity increases in drug loaded formulations. It has been also reported that reduced conductivity values may be attributed to phase separation and extend larger droplet radii [13].

Previously revealed pH of skin ranges of 5, 6, and 5.5 are considered to be average pH of the skin [14]. Results of pH analysis indicate that there is no variation in pH values of multiple emulsion samples kept in different conditions of storage. This may be attributed to stable conductivity values and absence of any chemical degradation. However, at 8°C and 40°C, the slight variation in pH observed may be due to slight chemical degradation while at 40°C this degradation may be due to production of acidic byproducts from the green tea extract as reported in previous work [14]. 

Results are also consistent with a previous study in which the stability of individual constituents of green tea extract was demonstrated at elevated temperatures (50°C and 80°C). In this study, emulsion systems were compared with solutions loaded with different green tea constituents and results were more promising for the emulsion systems than for the solutions [15]. 

Results of our study (Table 3) indicate that different storage conditions have no effect on pH and conductivity values; thus, no leakage appears and ultimately the formulations are stable regarding pH, globule size, and phase separation. Moreover, it is difficult to assess the emulsion stability solely by electrical conductivity.

### 3.3. Rheological Analysis

The consistency of the multiple emulsions, which is very important for cosmetic applications, can be evaluated using rheological methods. These methods can be applied for the primary as well as the final multiple emulsion, which may also contain a gel phase. Steady state shear stress-shear rate, constant stress, and oscillatory techniques may be applied. Most multiple emulsions are non-Newtonian and show pseudoplastic flow.

 Steady state shear stress-shear rate technique was applied to determine multiple emulsion flow parameters while fitting the data in Power low equation as we have not calculated yield stress:

(2) $$\begin{array}{c} \tau =K\gamma n, \end{array}$$

where *τ* is shear stress, *γ* is shear rate, *K* is consistency index, and *n* is flow behavior index.

 Consistency index *K* is a measure of the system consistency, and it is in relation with apparent viscosity. Flow behavior index *n* determines the degree of non-Newtonian behavior and varies in the range between 0 and 1. The non-Newtonian character of the investigated system is more pronounced for smaller values of constant *n* [16].

 Viscosities of multiple emulsion samples were measured at a speed of 100 to 200 rpm (with 20 increments) while applying shear rates from 200 to 400 (with 20 increments) on each sample. High shear rates were applied for quality assurance of emulsions by applying high shear stresses. All the multiple emulsions revealed non-Newtonian flow and shear thinning behavior on different conditions of storage. As the shear rate and shear stress increased, viscosity decreased. ANOVA one-way test described a significant reduction in the viscosity of emulsion sample kept at 40°C with 75% relative humidity. Grubbs' statistical test was applied to determine whether the most extreme value in the list was a significant outlier from the rest or not, but we found nonsignificant results for Grubbs' test. It was obvious that rheometer measurements were accurate; however, we were unable to find the reason behind reduced viscosity at 40°C with 75% relative humidity. There may be two reasons behind the reduction in viscosities: diffusion of the internal aqueous phase to the outer aqueous phase or rupturing of globules at high shear stresses. It is advisable to perform microscopy of samples after rheological analysis, so that bursting of globules may be observed. 

**Figure 2:**
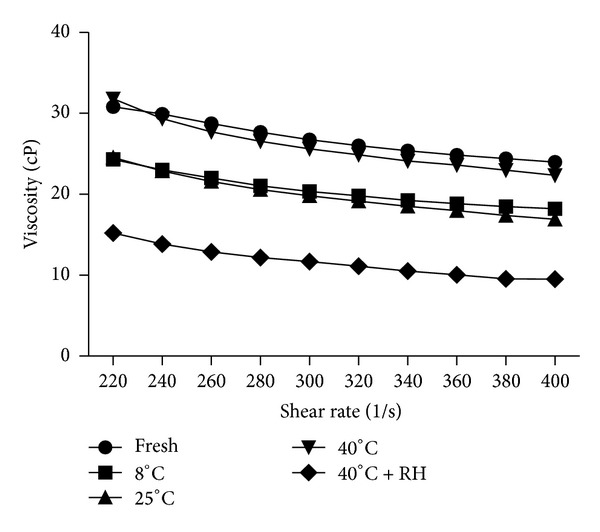
Viscosities of fresh and multiple emulsions kept at different storage conditions on varying shear rate.

 In our results, excellent fits were found to be 98.9 to 99.4 while the values of *K* and *n* are shown in Table 4. It was discovered that all samples exhibited pseudoplastic behavior with the flow behavior index (*n*) between 0.24 and 0.60. It was observed that the apparent viscosity decreased with increasing shear rate (11.66 to 26.86), and it was found that this tendency is more strongly exhibited in emulsions with higher emulsifier concentration and dispersed-phase volume. This rheological behavior could be described as typical for a non-Newtonian, pseudoplastic fluid [17]. It could be seen in Figure 2.

## 4. Conclusion

In current study, encapsulating 5% green tea extract in W/O/W emulsion, fabricated with two modern emulsifiers was tried. Promising stability characteristics in multiple emulsion samples kept in different storage conditions were observed. Emulsions samples were resistant to phase separation at exaggerated centrifugation test (ECT). A correlation between globule sizes of inner droplets and multiple droplets was found; however, different conditions of storage have no significant effect on globule sizes (*P* > 0.05). Without any pH adjustment, the pH value was within the limits of normal skin pH range. An inverse relationship was found between shear stress and viscosity data in all storage conditions. It is suggested that multiple emulsions loaded with 5% green tea extract have no substantial influence of varying storage conditions on its any of the physicochemical characteristics, but at 40°C with 75 relative humidity, viscosity of system dropped with the passage of time; thus, need may arise to add some gelling agent in the formulation to achieve long-term stability of the desired multiple emulsions at elevated temperatures so that formulation can be explored as a skin rejuvenating candidate.

## Figures and Tables

**Table 1:** Composition of green tea multiple emulsion.

	Ingredients	Composition (% w/w)
Primary emulsion (W/O)	Paraffin oil	25
	Abil EM90	5
	Green tea extract	5
	Magnesium sulfate	0.7
	Deionized water (Q.S)	100

Multiple emulsion (W/O/W)	Primary emulsion	80
	Brij 58	5
	Deionized water (Q.S)	100

W/O: water in-oil; Q.S.: quantum satis (as much as is enough); W/O/W: water in-oil-in-water.

**Table 2:** Mean globule size of 5% green tea multiple emulsions followed for 30 days.

Fresh	8°C	25°C	40°C	40°C with 75% RH
Globule size multiple droplets (*µ*m)				
10.29 ± 4.4	11.35 ± 3.8	12.77 ± 5.1	12.75 ± 5.9	11.22 ± 5.4

Globule size inner droplets (*µ*m)				
0.8 ± 0.4	1.4 ± 0.7	1.6 ± 0.5	1.6 ± 0.8	1.2 ± 0.6

Mean ± SD, (for multiple droplets *n* = 20 and for inner droplets *n* = 10).

**Table 3:** Centrifugation, mean conductivity, and pH values of 5% green tea multiple emulsions kept at different storage conditions.

Time	8°C	25°C	40°C	40°C with 75% RH
pH*				
24 h	5.13 ± 0.06	5.48 ± 0.03	5.31 ± 0.04	5.41 ± 0.02
48 h	5.11 ± 0.04	5.46 ± 0.35	5.12 ± 0.08	5.36 ± 0.24
07 d	5.70 ± 0.06	5.44 ± 0.07	5.02 ± 0.08	5.29 ± 0.16
15 d	5.55 ± 0.32	5.41 ± 0.36	5.05 ± 0.05	5.28 ± 0.08
30 d	5.56 ± 0.08	5.34 ± 0.06	5.09 ± 0.03	5.35 ± 0.09

Conductivity (*μ*S/cm)**				
24 h	14.2 ± 2.50	7.93 ± 1.10	9.70 ± 1.81	9.30 ± 1.59
48 h	14.7 ± 2.60	9.30 ± 2.95	9.90 ± 2.65	9.76 ± 2.01
07 d	15.3 ± 2.45	11.3 ± 1.81	10.8 ± 1.42	10.0 ± 2.05
15 d	15.5 ± 1.70	11.5 ± 1.15	11.4 ± 2.06	10.3 ± 0.50
30 d	15.6 ± 0.61	11.2 ± 0.78	9.37 ± 0.38	11.7 ± 0.81

Centrifugation/phase separation***				
24 h	S	S	S	S
48 h	S	S	S	S
07 d	S	S	S	S
15 d	S	S	S	S
30 d	S	S	S	S

*pH of fresh multiple emulsion = 5.31 ± 0.01; **conductivity of fresh multiple emulsion = 8.43 ± 0.21 *μ*S/cm; ***centrifugation at 5000 rpm for 20 minutes for each sample; S: stable.

**Table 4:** Results of rheological analysis followed for 30 days.

	Fresh	8°C	25°C	40°C	40°C RH
Consistency index (cP)	262.7	258.6	478.7	486.2	847.7
Flow index	0.60	0.55	0.44	0.48	0.24
Confidence of fit %	99.6	99.2	99.4	98.9	99.3
Apparent viscosity (cP)*	26.86	20.54	19.94	25.90	11.66

*Mean apparent viscosity at 100–200 rpm, RH = 75% relative humidity.
